# A *Burkholderia stabilis* outbreak associated with the use of ultrasound gel in multiple healthcare centres in Montréal, Canada, May–October 2021

**DOI:** 10.14745/ccdr.v49i78a03

**Published:** 2023-08-01

**Authors:** Christine Arsenault, Josée Harel, Florence Doualla-Bell, Yiorgos Alexandros Cavayas, Xavier Marchand-Sénécal, Charles Frenette, Yves Longtin, Linda Lalande, L Marie-Paule Diby, Nadia Desmarais

**Affiliations:** 1 Hôpital du Sacré-Cœur-de-Montréal, Montréal, QC; 2 Département de microbiologie, infectiologie et immunologie, Faculté de médecine, Université de Montréal, Montréal, QC; 3 Laboratoire de santé publique du Québec/Institut national de santé publique du Québec, Sainte-Anne-de- Bellevue, QC; 4 Département de médecine, Faculté de médecine, Université de Montréal, Montréal, QC; 5 Hôpital Maisonneuve-Rosemont, Montréal, QC; 6 McGill University Health Center, Montréal, QC; 7 Infectious Diseases Division, Faculty of Medicine and Health Sciences, McGill University, Montréal, QC; 8 Jewish General Hospital, Montréal, QC; 9 Service de prévention et contrôle des infections du CIUSSS du Nord de l’Ile de Montréal, Montréal, QC

**Keywords:** outbreak, *Burkholderia stabilis*, ultrasound gel, Canada

## Abstract

**Background:**

*Burkholderia stabilis* is a non-fermenting, gram-negative bacteria that has previously been implicated in multiple nosocomial outbreaks through the use of contaminated medical devices and substances. This article reports on an outbreak of *B. stabilis* infections and colonizations, involving 11 patients from five acute care hospitals in Montréal, Canada.

**Methods:**

One sample was not available for testing, but the remaining 10 isolates (91%) were sent for phylogenetic testing. Medical materials and the patients’ environments were also sampled and cultured. Samples were tested using pulsed field gel electrophoresis and multilocus sequence typing.

**Results:**

The outbreak was found to be associated with the use of intrinsically contaminated non-sterile ultrasound gel. Relatedness of the gel’s and the patients’ *B. stabilis* strains was demonstrated using gel electrophoresis and multilocus sequence typing analyses. The investigation was concluded with a prompt recall of the product, and the outbreak was declared over by the end of October 2021.

**Conclusion:**

Contaminated non-sterile gel caused infections and pseudo-infections in several patients.

## Introduction

On July 25, 2021, an unusually high number of requests for consultations (n=3) by the infectious diseases medical team were placed, seeking advice on *Burkholderia stabilis* bloodstream infections in the intensive care unit of the *Hôpital du Sacré-Coeur-de-Montréal*, a 440-bed teaching hospital in Montréal, Canada. It raised concerns about a possible outbreak and led to a formal investigation.

*Burkholderia stabilis* is a non-fermenting, oxidase-positive gram-negative bacillus, and is a ubiquitous environmental saprophyte. This member of the *B. cepacia* complex has been associated with nosocomial outbreaks of respiratory infections in patients with cystic fibrosis but can also cause non-respiratory infections in other populations through contamination of various medical devices. Washing gloves ((1)), chlorhexidine ((2)), alcohol-free mouthwash ((3)) and medication ((4)) have all been found to be sources of contamination in previous nosocomial outbreaks of *B. cepacia* complex. Although non-sterile, multi-use, ultrasound gel is appropriate for use on intact skin and on noncritical devices, it is known to support the growth of pathogenic bacteria ((5)) and has been associated with several outbreaks of *B. cepacia* complex in different settings ((6–8)).

The epidemiological investigation of the outbreak, the phylogenetic investigation, and the subsequent management are described to prevent further cases.

## Method

### Outbreak detection

On July 25, 2021, an outbreak of nosocomial bloodstream infections of an unknown source was suspected. An investigation was initiated by the infection control team to identify its source and to prevent exposure of additional patients. First, the laboratory information system was queried for previous positive culture specimens for either *B. stabilis* or *B. cepacia* complex. One previous positive blood culture (one or more bottle) for *B. stabilis* was identified on May 30, 2021, but the isolate had been discarded, in the meantime, as per laboratory protocol. This first case was considered as being part of the outbreak, although its isolate did not contribute to the analysis. Hence, a total of four patients were found to have at least one positive blood culture with *B. stabilis* over the course of six weeks, of which three isolates were available for further analysis. Three patients had their positive culture sampled 48 hours or more after admission, and one had a positive blood culture sampled on the day of admission. A preliminary case definition was established as a positive blood culture for *B. stabilis* sampled on the third day after hospital admission or later in the three-month period preceding July 25, 2021. This definition was used to be consistent with the case definition of a nosocomial bloodstream infection by the provincial surveillance program ((9)). Symptoms were not required to fit the case definition, and an infection diagnosis was not necessary for inclusion. When no symptom was attributed to the bacteria retrieved in a clinical specimen, it was considered either a contaminant or a colonizer. We defined a contaminant as an organism that is detected by culture but believed to be introduced in the process of sampling the bodily fluid or organ and absent in the fluid or organ itself. A colonizer is a saprophyte organism detected by culture but not causing disease.

A case definition for a possible case included any patient with a culture positive for *B. stabilis* or *B. cepacia* complex from any site (other than blood), either nosocomial or community-acquired in the three-month period preceding July 25, 2021.

### Investigations

First observations were conducted in the intensive care unit department, where all four cases had been identified. On July 29 and July 30, infection control practitioners audited diverse care techniques provided to patients and related procedures including bathing with single-use gloves, oral hygiene, use of thermometers, central venous catheter manipulations, use of sterile water, handling of multi-use ultrasound gel bottles, and disinfection of noncritical devices.

Subsequently, sampling of clean and sterile material was performed and sent for culture. Indwelling central catheter insertion sites were also swabbed. Sampled material included opened and sealed non-sterile ultrasound gel, sterile ultrasound gel, single-use commercial washing gloves, chlorhexidine wipes, sterile water and mouthwash.

Cultures were incubated on 5% blood sheep agar and MacConkey agar for 48 hours at 37°C in ambient air conditions. Morphologically compatible colonies were submitted for identification using the VITEK MS system using Database v3.1 (bioMérieux, France).

Pulse field gel electrophoresis and multilocus sequence typing analyses

Molecular typing analysis of *B. stabilis* isolates was done by pulse field gel electrophoresis (PFGE) and multilocus sequence typing (MLST) ((10,11)). Pulse field gel electrophoresis was carried out at the *Laboratoire de santé publique du Québec*. Sequence typing of *B. stabilis* isolates was performed by the National Laboratory of Microbiology of Canada according to the protocol and primers specified in a public database of MLST sequence data ((10–12)).

### Interventions

Positive cultures sampled from both opened and sealed ultrasound gel containers originating from the intensive care units were obtained on July 30. The use of all similar products was immediately discontinued at the intensive care units of the *Hôpital du Sacré-Coeur-de-Montréal* and affiliated hospitals. When additional positive cultures were obtained from ultrasound gel containers from other units, all gel bottles were discarded and replaced by an alternate product on August 2.

Montréal Public Health was notified on August 2 of a suspected contamination of ultrasound gel containers. A notice was sent to physicians and laboratories, and clinical specimens from other hospitals in the Montréal area were sent to the provincial public health laboratory.

Provincial health ministry was notified on August 4 and Health Canada was notified on August 6. A formal complaint was filed to the manufacturer on August 4 and the product was recalled the same day. To identify additional outbreak-related cases in other healthcare institutions in the province of Québec, a microbiology database search was conducted in several hospitals that used the same brand of ultrasound gel. Cultures from any sterile sites found to be positive with *B. stabilis* and *B. cepacia* complex were listed. The medical charts of patients with positive cultures were reviewed by local infectious disease consultants to determine whether the positive culture represented a true infection, a contaminant, or a colonization. Patients received care and antimicrobial treatment accordingly.

## Results

**Figure 1:**
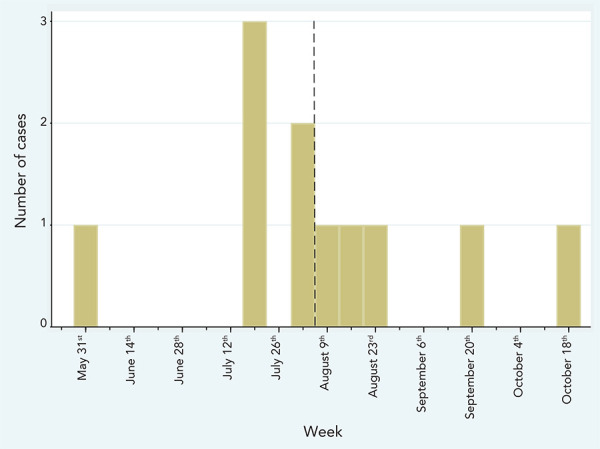
Cases of *Burkholderia stabilis* infections or pseudo-infections, by week of collection of first positive specimen, May–October 2021^a^ ^a^ Initial case reported on May 30^th^ is presumptively related to the outbreak, but no isolate was available for a pulse field gel electrophoresis (PFGE) and sequencing analysis. Dashed line shows date of product recall (August 4^th^). First day of the week is used as label

Over the course of the outbreak, a total of 11 cases of infections and pseudo-infections (detection of a colonizer or contaminant in a specimen sent for culture) were found in five Montréal hospitals, of which 10 isolates were available for analysis; eight specimens were collected between July 16 and August 24 and the last two were collected on September 20 and October 18, 2021 (**Figure 1**). The 11^th^ isolate had been discarded before the outbreak was declared.

The outbreak was considered over by the end of October 2021 as no further cases were reported and the identified source of the outbreak was no longer in operation.

The case definition used to initiate the investigation proved to be too restrictive, as specimens that were genetically related to the outbreak were sampled from both sterile and non-sterile sites. Consequently, the case definition was reviewed and updated on July 30 to include all cases of infection and pseudo-infections with a genetically related strain of *B. stabilis* recovered from any type of body specimen.

Of the 33 specimens sampled from medical material and the patients’ environment, six collected from different ultrasound gel bottles were positive for *B. stabilis*. Five of these bottles were factory sealed prior to sampling and one was already open and in use. *B. stabilis* was the only bacteria identified in culture. All other samples were negative. Isolates were sent to the public health reference laboratory for further analysis. All patients and gel isolates were clonal after sequencing analysis. All but one isolate was considered definitively related on PFGE analysis, displaying a unique PFGE pattern with restriction enzyme *Spel* (pulsovar A). One was considered likely related to the outbreak strains, exhibiting a closely related *Spel* pattern (pulsovar A2). In addition, all isolates shared the same MLST profile and were identified as MLST type ST51, confirming their relatedness.

No death has been attributed to an infection associated with this outbreak. While it is possible that medical care episodes were complicated by a positive blood culture, it was not possible to verify or quantify this impact. Patients presented with wide-ranging clinical profiles. The relevant clinical characteristics are reported in **Table 1**. Since no surgical site infection was reported, surgeries are not included in these reported data.

**Table 1:** Characteristics of patients with a positive culture with a clonal stain of *Burkholderia stabilis*

Hospital	Reason for admission	Procedures involving ultrasound gel prior to or at time of positive cultures	Type of specimens	Signification of culture result as per infectious disease consultant
1	Trauma	Central line insertion FAST ultrasound	Blood cultures	Infection
2	Cardiac arrest	Peripherally inserted central line, transthoracic echocardiogram	Endotracheal secretions	Colonization
2	Birth (newborn)	External fetal monitoring	Umbilical cord blood cultures	Contaminant
2	Fall	Peripherally inserted central line	Blood cultures	Infection
3	Trauma	Central line insertion FAST ultrasound	Blood cultures	Infection
3	Neurological condition	Peripherally inserted central line Venous cavography (using surface ultrasound)	Blood cultures	Infection
3	Neurological condition	Transthoracic cardiac ultrasound	Blood cultures	Infection
3	Trauma	Transthoracic cardiac ultrasound	Blood cultures	Infection
4	Orthopedic condition	Joint ultrasound	Synovial fluid	Contaminant
5	Congestive heart failure	Central line insertion, mesenteric angiogram and embolization, dialysis catheter insertion	Bronchoalveolar lavage	Infection

Abbreviation: FAST, Focused Assessment with Sonography in Trauma

## Discussion

This report documents an outbreak of *B. stabilis* associated with the use of contaminated non-sterile ultrasound gel. Ten clinical isolates and six isolates from opened and sealed ultrasound gel containers showed relatedness by PFGE and MLST analyses, supporting the hypothesis of ultrasound gel being the cause of the outbreak. Most patients were hospitalized in the intensive care unit, and many had a central venous line in place or were intubated.

A similar investigation reporting 119 cases of *B. stabilis* infections acquired from ultrasound gel produced by the same manufacturer was performed in the United States during the same period ((13)); the results of this investigation are consistent with our findings and support our conclusions.

In this outbreak, intrinsic product contamination occurred at the manufacturing stage, as demonstrated by the presence of bacterial strains in sealed gel bottles. *Burkholderia cepacia* complex organisms are frequently involved in recalls of non-sterile products ((14)). These bacteria are often resistant to biocides used to prevent bacterial proliferation and can survive for prolonged periods in low-nutrient environments. They are a frequent cause of pharmaceutical compound contamination, which can occur because of contaminated surfaces and materials, but most often through the inclusion of contaminated water ((14)). While non-sterile products are vulnerable to contamination, sterile products are manufactured in bacteria-free environments using sterile materials and are therefore much less likely to result in a contaminated product.

The exact mechanism allowing the non-sterile contaminated gel to lead to bacteremia remains unclear and is likely multifactorial. Visual audits did not reveal noncompliance to central line insertion standards ((15)) or non-critical devices disinfection ((16)), but it was noted that ultrasound gel was sometimes removed swiftly using a dry cloth after a bedside examination. However, these audits are by nature limited to a handful of observations. Although non-sterile ultrasound gel is to be used only on intact skin ((5)), similar outbreaks related to contaminated gel have occurred ((6–8)). We hypothesize that contamination of the intact skin of vulnerable patients leads to changes in the skin microbiome and colonization with *B. stabilis*. *Burkholderia stabilis* is more likely to be a causative organism if these colonized patients subsequently develop a healthcare-associated infection. This suggests sterile gel should be preferred before an impending invasive procedure, as a simple intervention that should reduce the likelihood of similar events.

Most cases occurred over a short period of time, but two cases phylogenetically related to the outbreak occurred after the month of August, with the latest on October 18. While it was not possible to prove this hypothesis, one likely explanation is that some gel bottles were not discarded immediately after the recall and were still in use at the time of the last case. Implementing a temporary prospective surveillance following a product recall could help address this situation and ensure containment of the outbreak.

One strength of this investigation was the fast identification of the source, leading to a prompt recall of the contaminated product. The causal relationship between the cases and the product is supported by the relatedness of the bacterial strains, demonstrated using multiple validated techniques. While it does not prove that the ultrasound gel caused all infections and pseudo-infections, it would be unlikely to observe such genetic similarity between bacteria retrieved in a product used on patients’ skin and cultures due to chance alone, considering the rarity of this pathogen in the infectious disease practice. Although enough isolates were retrieved to support the association, the earliest case’s isolate was not available to be analyzed. No systematic procedure was in place to refer *B. stabilis* isolates to the public health laboratory; therefore, our samples do not reflect the entire outbreak’s magnitude. We believed that enough data supported the association between the ultrasound gel and the positive cultures of clinical specimens, so no formal case-control study was performed.

This article describes an outbreak of infections and pseudo-infections with *B. stabilis*, attributed to intrinsically contaminated ultrasound gel. Non-sterile ultrasound gel is vulnerable to contamination with bacterial pathogens at the time of manufacturing, and from human cross-contamination after introduction into clinical use. Healthcare centres must remain aware of the potential for contamination of these products that could lead to multicenter outbreaks. The universal use of sterile single-use ultrasound gel containers could provide a theoretical advantage, but our study cannot determine whether switching to sterile gels could improve patient outcomes. Still, our study supports the generally accepted notion that single use sterile gels should be preferred over multiuse non-sterile gel in at-risk contexts, such as invasive procedures, procedures that involve sterile equipment and for procedures on mucous membranes or non-intact skin ((5)).
